# Supplementary material for: Complex‐centric proteome profiling by SEC‐SWATH‐MS
Source: Mol Syst Biol. 2019 Jan 14;15(1):e8438. doi: 10.15252/msb.20188438 (PMC6346213; doi:10.15252/msb.20188438)
Supplement: Supplementary file 8 — Dataset EV7 [file MSB-15-e8438-s008.zip › feature_plots_string/Q9UMX0.pdf]

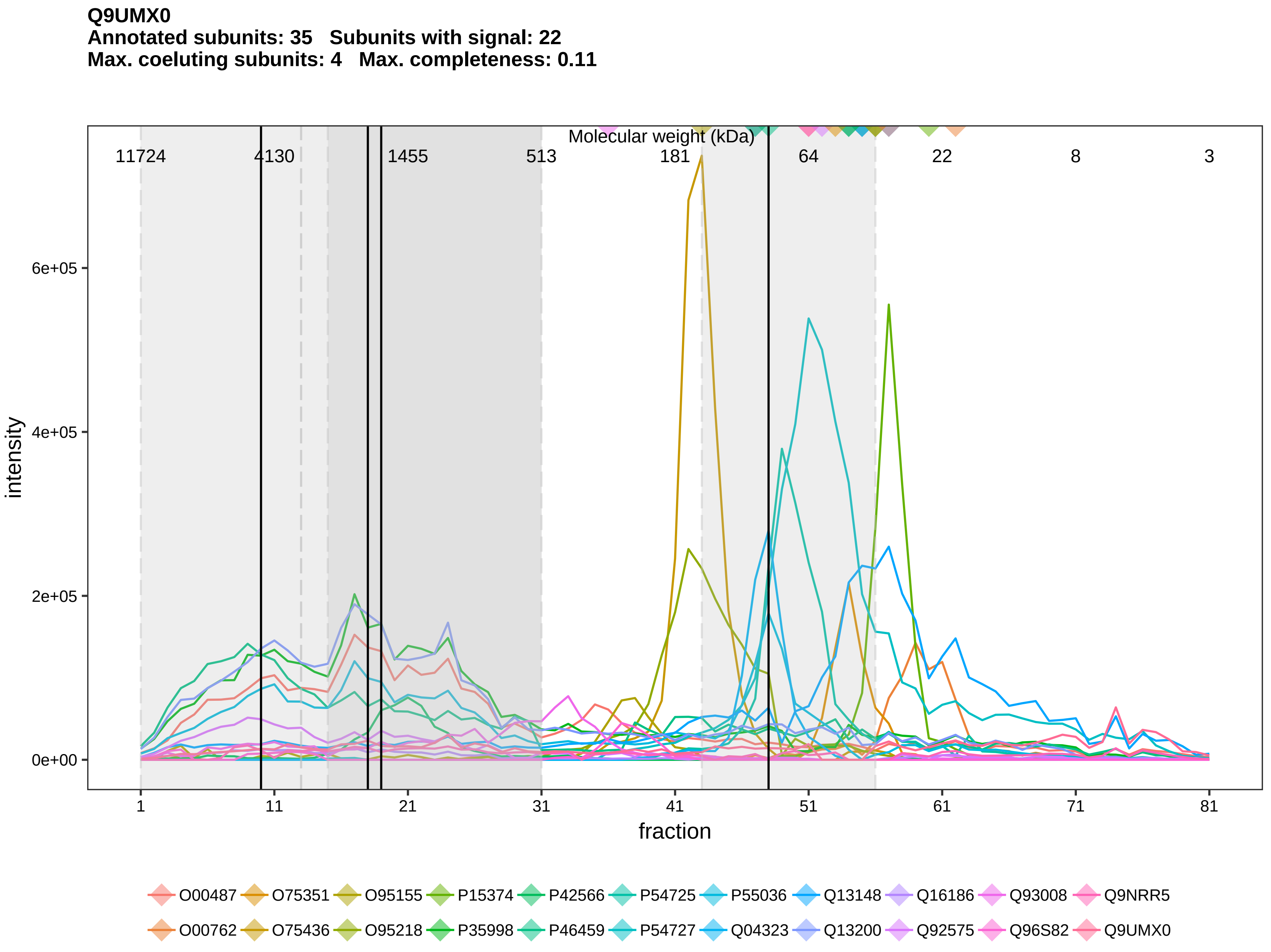

Q9UMX0
Annotated subunits: 35 Subunits with signal: 22
Max. coeluting subunits: 4 Max. completeness: 0.11
Molecular weight (kDa)
11724
4130
1455
513
181
64
22
8
3
intensity
6e+05
4e+05
2e+05
0e+00
1
11
21
31
41
51
61
71
81
fraction
O00487
O75351
O95155
P15374
P42566
P54725
P55036
Q13148
Q16186
Q93008
Q9NRR5
O00762
O75436
O95218
P35998
P46459
P54727
Q04323
Q13200
Q92575
Q96S82
Q9UMX0
